# Real-world outcomes of intranasal esketamine and intravenous ketamine induction therapy for treatment-resistant depression in a community clinic: a retrospective cohort study

**DOI:** 10.3389/fpsyt.2026.1867963

**Published:** 2026-07-09

**Authors:** Patrice A. Bellanti, Jordan Lewis, Brian Seifferth, Daniel Z. Adams

**Affiliations:** Ketamine Health and Wellness Clinics of Ohio, Dublin, OH, United States

**Keywords:** treatment-resistant depression, ketamine therapy for depression, intranasal esketamine, intravenous ketamine, comparative effectiveness, real-world community outcomes, outpatient psychiatric practice, antidepressant treatment failure

## Abstract

**Introduction:**

Intravenous (IV) ketamine and intranasal esketamine are NMDA receptor antagonists used for treatment-resistant depression (TRD). Both have demonstrated efficacy in controlled trials, but observational evidence from real-world community settings is limited.

**Methods:**

We conducted a single-center retrospective cohort study of adults aged 18 to 65 receiving induction therapy for TRD with intranasal esketamine or IV ketamine at a community psychiatric clinic from January 1 through December 31, 2025. Patients with prior exposure to either medication or to oral ketamine derivatives were excluded. The primary outcome was change in Patient Health Questionnaire-9 (PHQ-9) score from baseline to end of induction. Secondary outcomes included response (≥50% PHQ-9 reduction), remission (final PHQ-9 ≤4), clinically meaningful improvement (≥5 PHQ-9 reduction), induction completion, and adverse events. Within-group and per-protocol analyses were exploratory.

**Results:**

Sixty-three patients met inclusion criteria (esketamine n=37; IV ketamine n=26). Baseline PHQ-9 scores were similar (18.22±4.49 vs. 18.27±5.41; P = 0.967), as was mean change from baseline (-10.31±5.59 vs. -9.50±5.69; mean difference 0.81, 95% CI -2.12 to 3.75; P = 0.589). Response rates were 64.9% vs. 69.2% (RR 0.94, 95% CI 0.66 to 1.33; P = 0.790), remission 32.4% vs. 23.1% (RR 1.41, 95% CI 0.61 to 3.26; P = 0.573), and clinically meaningful improvement 83.8% vs 73.1% (RR 1.15, 95% CI 0.87 to 1.51; P = 0.353). Induction completion exceeded 90% in both groups; one patient per cohort discontinued from intolerable side effects, and no serious adverse events occurred. Within-group PHQ-9 reduction was large: 10.31±5.59 points (paired t[34]=10.91; P<0.001; Cohen’s d=1.84) for esketamine and 9.50±5.69 points (paired t[23]=8.18; P<0.001; Cohen’s d=1.67) for ketamine. Findings remained statistically significant and clinically large under a pre-specified baseline observation carried forward (BOCF) sensitivity analysis (Cohen’s d=1.65 and 1.45).

**Conclusions:**

Induction therapy with intranasal esketamine and intravenous ketamine was associated with robust antidepressant effects in a community outpatient setting, consistent with prior trial data. The modest sample size limits power to detect between-group differences and increases the risk of type II error; the absence of statistically significant between-group differences should therefore not be interpreted as evidence of equivalence. Protocol asymmetry between arms (esketamine: 12 sessions over 8 weeks; IV ketamine: 6 sessions over 3 weeks) further limits direct comparison of endpoint values between groups. Practical considerations including insurance coverage, cost, and administration logistics may help guide treatment selection. Longitudinal follow-up is planned to characterize treatment durability.

## 1 Introduction

Major depressive disorder (MDD) is among the leading causes of disability worldwide, contributing substantially to global disease burden. Of those suffering from MDD, approximately one-third fail to achieve remission with first-line antidepressant therapies. A substantial proportion of these patients remain symptomatic despite multiple pharmacological interventions, a condition commonly referred to as treatment-resistant depression (TRD) (1). Patients with TRD experience increased morbidity, functional impairment, healthcare utilization, and suicide risk compared to those whose depression responds to standard treatments (2).

Conventional antidepressant medications primarily target monoaminergic neurotransmitter systems and can require several weeks to produce clinically meaningful improvement (3). Large observational and clinical trial datasets show that nearly half of patients do not respond to a first antidepressant trial, and the odds of success decline with each subsequent medication trial failure (4, 5). This has led to increased interest in alternative medications targeting different mechanisms. Ketamine, an N-methyl-D-aspartate (NMDA) receptor antagonist that modulates glutamatergic neurotransmission and was originally developed as an anesthetic, has emerged over the past two decades as a novel treatment for TRD with rapid antidepressant effects (6). In a landmark randomized controlled trial, a single intravenous infusion of ketamine produced rapid and significant antidepressant effects in patients with TRD compared to placebo (7). Subsequent studies have demonstrated that ketamine infusions can produce and sustain antidepressant response beyond that of traditional antidepressants and reduce suicidal ideation in patients with severe depression (8).

Additional trials have validated the efficacy of intravenous ketamine infusions for TRD. However, it remains off-label and is often not reimbursed by insurance. Intranasal esketamine was subsequently introduced as an FDA-approved alternative. Esketamine is the S-enantiomer of ketamine, developed as an intranasal formulation to allow administration in outpatient psychiatric settings. Randomized clinical trials have repeatedly demonstrated that intranasal esketamine produces meaningful reduction in depressive symptoms for patients with TRD compared to standard therapies alone (9, 10). These findings led to regulatory approval of esketamine nasal spray for TRD in the United States and other countries. Important questions remain regarding the relative effectiveness and tolerability between the two formulations, particularly outside of controlled clinical trial environments.

Currently, the majority of evidence comparing ketamine and esketamine has been derived from randomized clinical trials conducted in specialized research settings with carefully selected populations. While these studies provide critical information about efficacy, their findings may not fully reflect treatment outcomes in routine psychiatric practice, where patient populations are often more heterogeneous with multiple psychiatric and medical co-morbidities and varied adherence patterns (11). Observational studies conducted in community treatment settings therefore provide valuable complementary evidence regarding real-world effectiveness, tolerability, and patterns of treatment discontinuation. Real-world cohorts evaluating both treatments may further clarify whether the antidepressant effects observed in controlled trials translate to community psychiatric settings and whether differences in route of administration, treatment protocols, or patient selection influence clinical outcomes.

The following study aimed to describe real-world effectiveness of intranasal esketamine and intravenous ketamine induction therapy in a community psychiatric clinic using standardized protocols previously reported in the literature. Changes in depressive symptom severity measured by the Patient Health Questionnaire-9 (PHQ-9) were used between and within groups to compare the cohorts as a validated measure of depression severity that has demonstrated excellent internal consistency and sensitivity to treatment-related symptom change in both research and clinical practice (12). In this manuscript, the term “induction therapy” refers to the initial course of ketamine or esketamine treatments delivered over a defined period to establish an antidepressant response prior to any subsequent maintenance dosing, consistent with usage in pivotal trial literature (9, 10, 16, 17).

## 2 Methods

### 2.1 Study design and oversight

This was a single-center retrospective observational cohort study of patients with TRD presenting to our community mental health clinic for induction therapy with either intranasal esketamine or intravenous ketamine from January 1, 2025 through December 31, 2025. This retrospective chart review was reviewed and approved by an independent IRB (IRB ID: 2026-0152; approved March 18, 2026). A waiver of HIPAA authorization was granted under 45 CFR 164.512(i) (2)(ii) given the minimal-risk nature of the research and impracticability of obtaining individual consent. TRD was defined as failure of ≥2 adequate trials of antidepressants from different pharmacological classes during the current depressive episode, consistent with contemporary field standards (adequate defined as ≥6 weeks at a minimum therapeutic dose). The design pre-specified adults ages 18 to 65 without prior exposure to either medication or oral ketamine derivative meeting the aforementioned strict criteria for TRD, with a focus on PHQ-9 change over the induction period as the primary end point. Patients over 65 years of age were excluded specifically to avoid confounding from neurocognitive co-morbidities that increase with aging. Other exclusion criteria included pregnancy, no-show status, social issues impacting ability to receive treatment, insurance change affecting the treatment course, change in treatment center, or relocating during the course of therapy.

### 2.2 Data source and cohort assembly

Data were abstracted from the clinic’s electronic medical record using a standardized review method. Patients were included only if they met eligibility criteria previously noted and specifically having failed ≥2 adequate trials of antidepressants from different pharmacological classes during the current depressive episode. Eligible patients were identified through direct chart review, with the source dataset containing demographics, serial PHQ-9 values by treatment session, reason for discontinuation if applicable, adverse events documented throughout therapy if they occurred, and systematic review of medical documentation for completeness. Specifically, adverse events leading to treatment discontinuation were sought, such as severe hypertension, intolerable central nervous system or gastrointestinal side effects, emergency department visitation, or hospitalization.

The retrospective design and the structure of the source EMR limited the granularity of certain baseline variables. Detailed prior antidepressant trial counts beyond the categorical ≥2-failure TRD-eligibility threshold, duration of current depressive episode, and comprehensive concomitant medication lists were not consistently captured in a structured, abstractable format across the cohort. These variables are acknowledged as unmeasured in the limitations.

### 2.3 Treatment protocols and outcomes

Induction protocols reflected routine practice following published recommendations.

#### 2.3.1 Intranasal esketamine protocol

Intranasal esketamine was administered at a starting dose of 56 mg and up-titrated as tolerated to 84 mg twice weekly for 4 weeks followed by once weekly for 4 weeks, for a total of 12 induction treatments. All esketamine treatments were administered in a Spravato REMS-certified outpatient setting in accordance with the Risk Evaluation and Mitigation Strategy program (13). Patients self-administered the nasal spray under direct supervision of a healthcare provider. Per REMS requirements, blood pressure was measured prior to dosing, at approximately 40 minutes post-dose, and at two hours. Additionally, patients were monitored for at least two hours after administration for sedation, dissociation, respiratory depression, and changes in heart rate, and respiratory status including pulse oximetry during these same time intervals and as indicated. Patients were not discharged until a healthcare provider determined they were clinically stable, and all patients were required to arrange transportation home and to avoid driving or operating machinery until the following day after restful sleep. A Patient Monitoring Form was completed and submitted within seven days for every session as required by the Spravato® REMS program.

#### 2.3.2 Intravenous ketamine protocol

Intravenous ketamine was administered at 0.5 mg/kg as a continuous infusion over 40 minutes, given twice weekly for 3 weeks for a total of 6 induction treatments. Vital signs (blood pressure, heart rate, and peripheral oxygen saturation) were obtained immediately prior to infusion, at the 20-minute midpoint of the infusion, and at the conclusion of the infusion using a standard upper-arm sphygmomanometer cuff. Heart rate and peripheral oxygen saturation (SpO2) were monitored continuously throughout the infusion using bedside finger-based pulse oximetry. Continuous monitoring data were observed in real time by a clinician. A licensed clinician was physically present with the patient throughout the infusion and post-infusion observation. Following infusion completion, patients were observed for a minimum of one hour or longer until return to clinical baseline as determined by the supervising clinician. As with esketamine, all patients were required to arrange transportation home and were counseled to avoid driving or operating machinery for the remainder of the treatment day.

#### 2.3.3 Common safety procedures and side-effect management

Both treatment modalities used a standardized clinic protocol for management of treatment-emergent nausea, which is among the more common adverse effects of NMDA antagonist administration. Ondansetron 4–8 mg was available in oral or intravenous form for symptomatic treatment of nausea during or after either treatment, and clinicians were permitted to administer ondansetron prophylactically prior to dosing in patients with a documented history of treatment-related nausea. Transient elevations in blood pressure were managed with continued observation; per the standing clinic protocol, persistent systolic blood pressure ≥180 mmHg or diastolic ≥110 mmHg would prompt consultation with a clinician experienced in blood pressure management, with medications including oral clonidine and hydralazine available at the consultant’s request. However, no patient in this cohort required such consultation and no serious adverse events were documented during the study period. Both arms received clinical support from nursing or supervising clinical staff at each session. No concurrent formal psychotherapy was co-administered in either arm during the induction period. The substantially greater number of structured clinical contacts in the esketamine arm (12 sessions with REMS-mandated provider monitoring) relative to the IV ketamine arm (6 sessions) represents a meaningful difference in total therapeutic contact time that may carry non-specific psychosocial benefit independent of pharmacological effects, an implementation consideration recognized in the esketamine delivery literature (25).

#### 2.3.4 Outcomes

The study was intentionally restricted to induction therapy and did not evaluate durability or maintenance outcomes, which are planned for a future analysis. The primary outcome was change in PHQ-9 score from baseline to the last observed induction PHQ-9 value. Secondary outcomes included response (≥50% reduction in PHQ-9 from baseline), remission (final PHQ-9 ≤4), clinically meaningful improvement (≥5 point absolute reduction in PHQ-9), induction completion, and treatment emergent adverse events. Serious safety events as previously described were systematically sought. The first PHQ-9 (baseline) was recorded prior to induction and the last PHQ-9 (final) value was recorded at the end of induction within 30 days of the final treatment.

The two induction protocols intentionally reflect their respective evidence-based clinical practice standards rather than an experimentally harmonized schedule. While this design maximizes ecological validity and reflects the protocols community providers actually employ, it introduces meaningful asymmetry in total treatment sessions (12 vs. 6) and total induction duration (8 vs. 3 weeks). The longer esketamine induction may contribute to its treatment trajectory through both cumulative pharmacodynamic effects and the passage of time, and the present analysis does not isolate drug-specific effects from protocol duration effects. This limitation is discussed in detail below.

### 2.4 Statistical analysis

Continuous variables are reported as means with standard deviations and compared using Welch’s t-test. Categorical variables were compared with Fisher’s exact test given modest sample sizes. Between-group effect sizes for continuous outcomes were expressed as mean differences with 95% confidence intervals (CI). Effect sizes for binary outcomes were expressed as risk ratios (RR). Race and ethnicity were summarized descriptively given sparse category counts precluding meaningful inferential testing.

Missing data were handled differently for binary and continuous outcomes. For binary outcomes in the primary analytic cohort (response, remission, and clinically meaningful improvement), every patient with a valid baseline PHQ-9 score was included in the denominator regardless of whether they completed treatment, including those who dropped out due to perceived non-response or because of side effects. This conservative approach follows intention-to-treat principles and avoids the need to impute missing PHQ-9 scores. For continuous outcomes (mean PHQ-9 change and within-group paired comparisons), the analysis included only patients with both a baseline and a final PHQ-9 score (complete-case analysis).

To quantify the influence of missing end-of-induction PHQ-9 data on continuous outcome estimates, a pre-specified baseline observation carried forward (BOCF) sensitivity analysis was performed. In this analysis, each patient missing an end-of-induction PHQ-9 had their baseline score substituted as their final score, equivalent to assuming zero within-person change. BOCF analysis was selected deliberately as the most conservative continuous-outcome imputation: by assigning zero improvement to non-completers, it produces effect-size estimates that cannot be inflated by missing-data assumptions favorable to the treatment. We acknowledge that BOCF can introduce a conservative bias and that alternative approaches such as multiple imputation or mixed-effects models for repeated measures (MMRM) could yield modestly larger effect estimates; we selected BOCF specifically because the goal of the sensitivity analysis was to test whether within-group findings would hold under the least favorable assumption regarding dropouts, not to maximize estimated effect sizes. BOCF was chosen in preference to last observation carried forward (LOCF), which is increasingly disfavored in psychiatric trials due to its tendency to bias estimates when dropout is related to treatment response. For the within-group analysis, the change in PHQ-9 from baseline to end of induction was assessed using paired t-tests for each treatment group independently. Within-group effect sizes were expressed as Cohen’s d.

Finally, a per-protocol analysis looking at rates of response, remission, and clinically meaningful improvement was performed to further characterize differences between cohorts among patients completing all planned sessions. A two-sided significance threshold of α = 0.05 was applied throughout. The study was not powered to detect between-group differences. Given the modest sample size, the study has limited statistical power to detect clinically meaningful between-group differences, and non-significant between-group findings should be interpreted in the context of substantial risk of type II error. No formal adjustment for multiple comparisons was performed, as the primary outcome was pre-specified and secondary outcomes are reported as hypothesis-generating within a descriptive retrospective cohort design. Findings from secondary and per-protocol analyses should therefore be interpreted accordingly.

## 3 Results

### 3.1 Cohort characteristics

**Figure 1:**
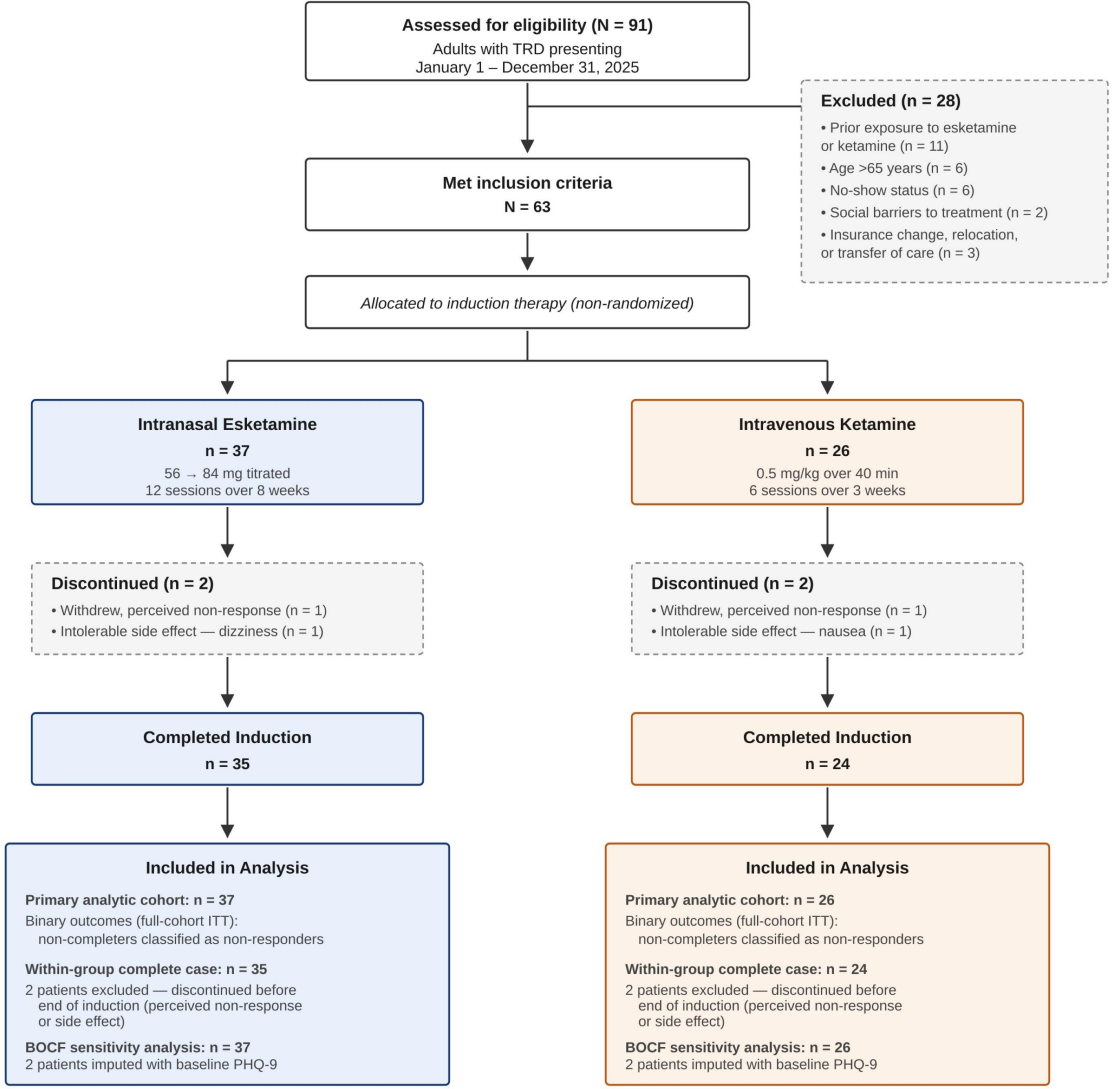
Participant flow and analytic cohort definitions. CONSORT-style flow diagram depicting participant screening, eligibility assessment, treatment allocation, and analytic cohort derivation. Of 91 adults with treatment-resistant depression assessed for eligibility between January 1 and December 31, 2025, 28 were excluded and 63 met inclusion criteria. Allocation to intranasal esketamine (n = 37; 56-84 mg titrated, 12 sessions over 8 weeks) or intravenous ketamine (n = 26; 0.5 mg/kg over 40 minutes, 6 sessions over 3 weeks) was non-randomized. Each cohort experienced two early discontinuations. Three analytic cohorts are defined for each arm: the primary analytic cohort (intent-to-treat aligned; non-completers classified as non-responders), the within-group complete-case cohort (completers only), and the BOCF sensitivity analysis cohort (non-completers imputed with baseline PHQ-9).

Of 91 patients assessed for eligibility during the study period, 28 were excluded prior to induction (11 with prior exposure to esketamine or ketamine, 6 over 65 years of age, 6 with no-show status, 2 with social barriers to treatment, 1 who changed insurance and did not complete treatment, 1 with relocation, and 1 with transfer of care to another center). A total of 63 patients met criteria for the primary analysis: 37 in the intranasal esketamine group and 26 in the intravenous ketamine group. The intranasal esketamine cohort comprised 35 patients completing the full induction course, with 1 patient who withdrew because of perceived non-response and 1 patient who discontinued because of side effects (intolerable dizziness). The intravenous ketamine cohort comprised 24 patients completing the full induction course, with 1 patient who withdrew because of perceived non-response and 1 patient who discontinued because of side effects (intolerable nausea). Baseline age, sex distribution, and PHQ-9 severity were similar between groups (**Table 1**). A patient flow diagram summarizing screening, exclusions, treatment assignment, completion, and missing end-of-induction PHQ-9 data is provided in **Figure 1**.

**Table 1:** Baseline characteristics of the primary analytic cohort.

Characteristic	Intranasal esketamine (n=37)	Intravenous ketamine (n=26)	Mean Difference	95% CI	P value
Age, years	41.24 ± 13.28	39.92 ± 10.45	1.32	-4.55 to 7.19	0.661
Male sex, n (%)	19 (51.4)	10 (38.5)	–	–	0.442
Female sex, n (%)	18 (48.6)	16 (61.5)	–	–	–
Race or ethnicity, n (%)			–		
*Caucasian*	31 (83.8)	22 (84.6)	–	–	–
*African American*	2 (5.4)	1 (3.8)	–	–	–
*Asian*	2 (5.4)	2 (7.7)	–	–	–
*Hispanic/Latino*	1 (2.7)	1 (3.8)	–	–	–
*Middle Eastern*	1 (2.7)	0 (0.0)	–	–	–
Baseline PHQ-9 score	18.22 ± 4.49	18.27 ± 5.41	-0.05	-2.59 to 2.48	0.967

Data are mean ± SD or n (%) unless otherwise indicated. Age and baseline PHQ-9 were compared using Welch’s t-test; sex compared using Fisher’s exact test. Race and ethnicity are reported descriptively; sparse category counts precluded meaningful inferential testing.

### 3.2 Primary and secondary outcomes

**Figure 2:**
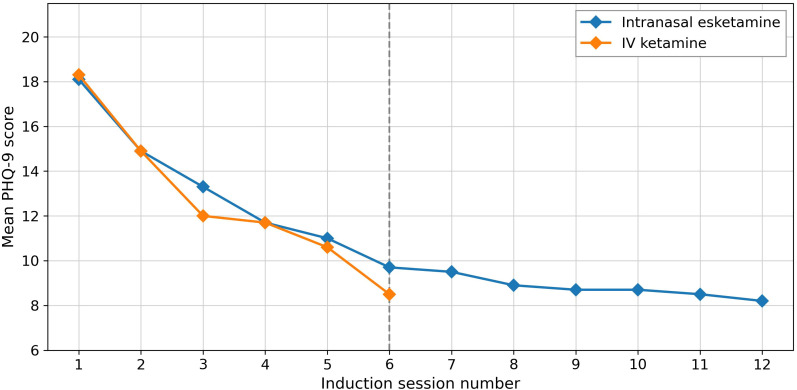
Mean PHQ-9 trajectory during induction. Mean PHQ-9 scores plotted by induction session for each treatment cohort. Intranasal esketamine trajectories extend through session 12 (8 weeks); intravenous ketamine trajectories terminate at session 6 (3 weeks) per protocol. The vertical dashed reference line at session 6 delineates the end of the intravenous ketamine induction course and enables a time-matched interim comparison: PHQ-9 in the esketamine cohort at session 6 was approximately 9.7 versus 8.5 in the ketamine cohort at end of induction. Trajectory data beyond session 6 reflect the esketamine arm only and are not interpretable as a between-group comparison, given the prespecified protocol difference in session count (12 vs. 6 induction sessions).

Mean baseline PHQ-9 scores were 18.22 ± 4.49 in the intranasal esketamine group and 18.27 ± 5.41 in the intravenous ketamine group (mean difference -0.05, 95% CI -2.59 to 2.48, P = 0.967). Mean final PHQ-9 scores were 8.17 ± 5.66 and 8.50 ± 6.33 respectively (mean difference -0.33, 95% CI -3.48 to 2.82, P = 0.839). Mean change in PHQ-9 during induction was -10.31 ± 5.59 with intranasal esketamine and -9.50 ± 5.69 with intravenous ketamine (mean difference 0.81, 95% CI -2.12 to 3.75, P = 0.589). The mean clinical PHQ-9 trajectory during induction is shown in **Figure 2**.

**Figure 3:**
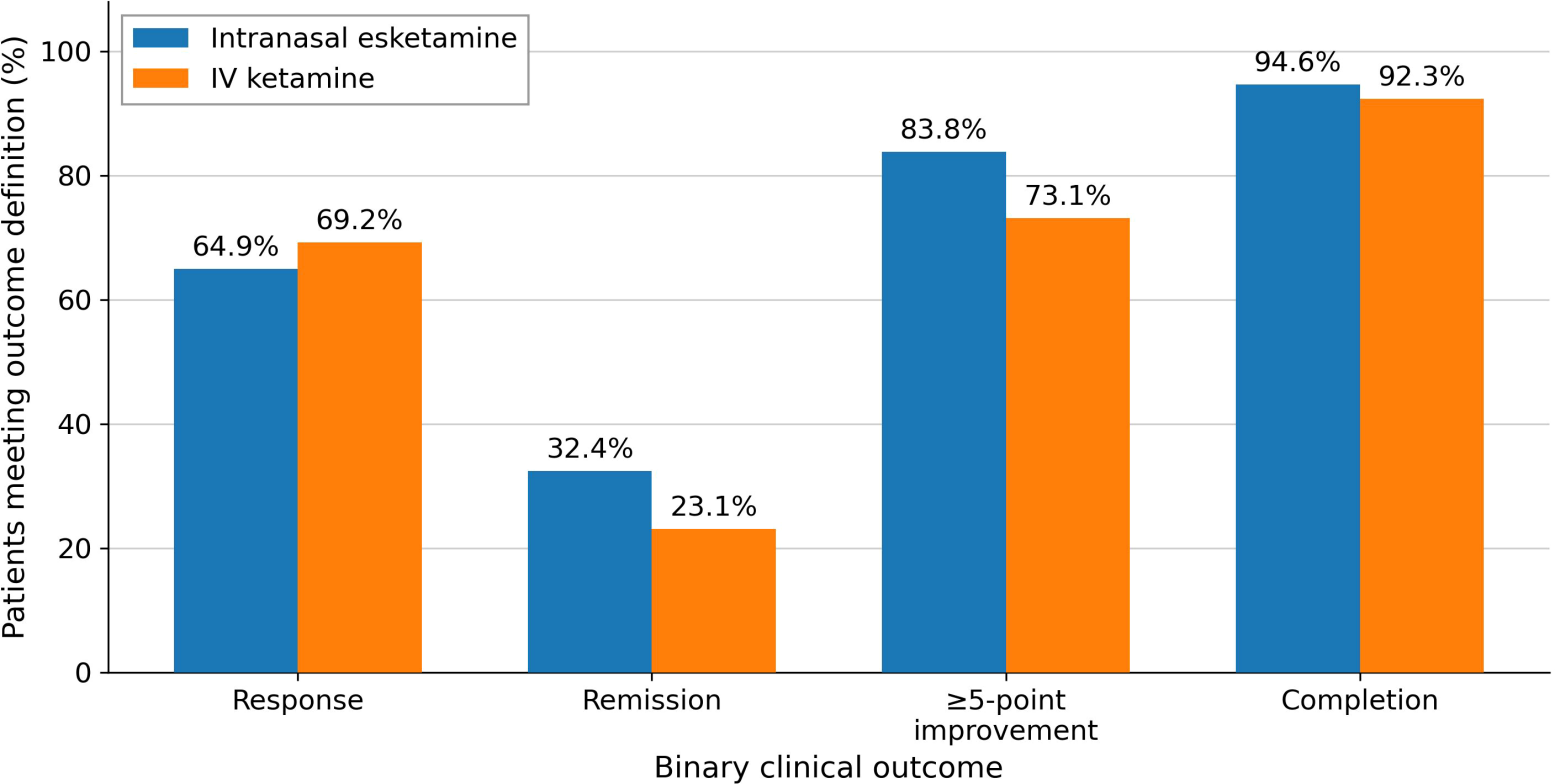
Binary clinical outcomes in the primary analytic cohort. Bars represent the percentage of patients in each treatment group meeting each prespecified binary outcome definition, with denominators from the full primary analytic cohort (intranasal esketamine n = 37; intravenous ketamine n = 26); non-completers were classified as non-responders. Outcome definitions-Response: ≥50% reduction in PHQ-9 from baseline; Remission: final PHQ-9 ≤4; Clinically meaningful improvement: ≥5-point reduction in PHQ-9 from baseline; Completion: completion of the full prespecified induction protocol. No statistically significant between-group differences were observed for any outcome (all p > 0.05, Fisher's exact test). The study was not powered to detect between-group differences; observed differences are reported descriptively.

Within the esketamine cohort, the mean PHQ-9 score at session 6 was approximately 9.7, indicating comparable interim effect to the intravenous ketamine cohort’s end-of-induction mean of 8.5, with further incremental reduction observed between sessions 6 and 12 in the esketamine arm. Categorical outcomes were likewise similar between groups (**Table 2**). Response occurred in 24 of 37 patients (64.9%) in the intranasal esketamine group and 18 of 26 patients (69.2%) in the intravenous ketamine group (RR 0.94, 95% CI 0.66 to 1.33, P = 0.790). Clinically meaningful improvement occurred in 31 of 37 (83.8%) and 19 of 26 (73.1%) of patients respectively (RR 1.15, 95% CI 0.87 to 1.51, P = 0.353). Remission occurred in 12 of 37 (32.4%) and 6 of 26 (23.1%) of patients (RR 1.41, 95% CI 0.61 to 3.26, P = 0.573). Completion exceeded 90% in both groups. One side effect discontinuation occurred in each group (“severe dizziness” and “severe nausea”), both leading to discontinuation, and no serious safety events were documented. **Figure 3** depicts the binary outcomes in the primary analytic cohort, with bars representing the percentage of patients meeting each outcome definition within each treatment group.

**Table 2:** Primary and secondary outcomes in the primary analytic cohort.

Outcome	Intranasal esketamine	Intravenous ketamine	Mean Difference or RR	95% CI	P value
Continuous outcomes (complete case)					
PHQ-9 mean reduction, baseline to induction completion	-10.31 ± 5.59 (n=35)	-9.50 ± 5.69 (n=24)	Δ 0.81	-2.12 to 3.75	0.589
Final PHQ-9 score, mean ± SD	8.17 ± 5.66 (n=35)	8.50 ± 6.33 (n=24)	Δ -0.33	-3.48 to 2.82	0.839
Binary outcomes (full cohort)					
Response	24/37 (64.9)	18/26 (69.2)	RR 0.94	0.66 to 1.33	0.790
Remission	12/37 (32.4)	6/26 (23.1)	RR 1.41	0.61 to 3.26	0.573
Clinically meaningful improvement	31/37 (83.8)	19/26 (73.1)	RR 1.15	0.87 to 1.51	0.353
Completed induction	35/37 (94.6)	24/26 (92.3)	RR 1.02	0.90 to 1.17	1.000
Discontinued due to side effects	1/37 (2.7)	1/26 (3.8)	–	–	1.000
Serious safety events	0 (0.0)	0 (0.0)	–	–	–

Data are mean ± SD or n/total (%) unless otherwise indicated. Binary outcomes use a full-cohort denominator; all patients with a valid baseline PHQ-9 remain in the denominator. Continuous outcomes reported on a complete-case basis with n shown.

### 3.3 Within-group primary analysis - complete case

The primary within-group analysis demonstrated large, statistically significant reductions in PHQ-9 from baseline to end of induction in both treatment groups. In the intranasal esketamine cohort (n=35 complete-case pairs), mean PHQ-9 decreased from 18.49 ± 4.27 to 8.17 ± 5.66, corresponding to a mean reduction of -10.31 ± 5.59 points (95% CI 8.46 to 12.17, paired t[34]=10.91, P<0.001, Cohen’s d = 1.84). In the intravenous ketamine cohort (n=24 complete-case pairs), mean PHQ-9 decreased from 18.00 ± 5.45 to 8.50 ± 6.33, corresponding to a mean reduction of -9.50 ± 5.69 points (95% CI 7.22 to 11.78, paired t[23]=8.18, P<0.001, Cohen’s d = 1.67). Complete-case baseline values differ slightly from **Table 1** total given two patients per cohort who did not complete induction were excluded from the paired analysis. These primary within-group results are summarized in **Table 3**.

**Table 3:** Primary within-group analysis (complete case).

	Intranasal esketamine (n=35)	Intravenous ketamine (n=24)
Baseline PHQ-9, mean ± SD	18.49 ± 4.27	18.00 ± 5.45
Final PHQ-9, mean ± SD	8.17 ± 5.66	8.50 ± 6.33
Mean PHQ-9 reduction, mean ± SD	-10.31 ± 5.59	-9.50 ± 5.69
Paired t-statistic (df)	10.91 (df=34)	8.18 (df=23)
Within-group P value	< 0.001	< 0.001
95% CI for mean reduction	8.46 to 12.17	7.22 to 11.78
Cohen’s d (paired)	1.84	1.67
Between-group mean difference	0.81 (95% CI -2.12 to 3.75), P = 0.589	

Complete-case paired analysis. Patients missing end-of-induction PHQ-9 are excluded from this table.

### 3.4 Within-group BOCF sensitivity analysis

The pre-specified BOCF sensitivity analysis demonstrated statistically significant reductions in PHQ-9 in both groups. In the intranasal esketamine group (n=37, 2 imputed), mean PHQ-9 decreased from 18.22 ± 4.49 to 8.46 ± 5.78, corresponding to a mean reduction of -9.76 ± 5.93 points (95% CI 7.85 to 11.67, paired t[36]=10.01, P<0.001, Cohen’s d = 1.65). In the intravenous ketamine group (n=26, 2 imputed), mean PHQ-9 decreased from 18.27 ± 5.41 to 9.50 ± 7.10, corresponding to a mean reduction of -8.77 ± 6.04 points (95% CI 6.45 to 11.09, paired t[25]=7.41, P<0.001, Cohen’s d = 1.45). Under BOCF, mean PHQ-9 was attenuated by 0.56 points in the intranasal esketamine cohort and 0.73 in the intravenous ketamine cohort relative to the primary complete-case estimates, with corresponding Cohen’s d reductions of 0.20 and 0.22 respectively. Both within-group paired comparisons remained highly statistically significant (P<0.001), both effect sizes remained in the large range (d ≥ 0.8), and the direction and statistical significance of the between-group comparisons of change scores are unchanged (**Table 4**).

**Table 4:** Within-group BOCF sensitivity analysis (full cohort).

	Intranasal esketamine (n=37)	Intravenous ketamine (n=26)
Baseline PHQ-9, mean ± SD	18.22 ± 4.49	18.27 ± 5.41
Final PHQ-9, mean ± SD	8.46 ± 5.78	9.50 ± 7.10
Mean PHQ-9 reduction, mean ± SD	-9.76 ± 5.93	-8.77 ± 6.04
Paired t-statistic (df)	10.01 (df=36)	7.41 (df=25)
Within-group P value	< 0.001	< 0.001
95% CI for mean reduction	7.85 to 11.67	6.45 to 11.09
Cohen’s d (paired)	1.65	1.45
Between-group mean difference	0.99 (95% CI -2.02 to 3.99), P = 0.515	

The BOCF (baseline observation carried forward) sensitivity analysis replaces each missing end-of-induction PHQ-9 with the patient’s baseline score, equivalent to assuming no within-person change for patients who did not complete induction therapy. BOCF is the most conservative continuous-outcome imputation and is reported to quantify robustness of the primary conclusions.

### 3.5 Per-protocol analysis

In the per-protocol analysis (**Table 5**), response rates were similar (68.6% vs. 75.0%, RR 0.91, 95% CI 0.66 to 1.26, P = 0.771), as were rates of remission (34.3% vs. 25.0%, RR 1.37, 95% CI 0.60 to 3.15, P = 0.568) and clinically meaningful improvement (88.6% vs. 79.2%, RR 1.12, 95% CI 0.88 to 1.42, P = 0.464). Per-protocol results are directionally consistent with the primary analysis and are reported for completeness. As an exploratory analysis subject to informative dropout bias, they should not be interpreted as the primary estimate of treatment efficacy.

**Table 5:** Exploratory per-protocol analysis.

Outcome	Intranasal esketamine (n=35)	Intravenous ketamine (n=24)	RR (95% CI)	P value
Response, n (%)	24 (68.6)	18 (75.0)	0.91 (0.66 to 1.26)	0.771
Remission, n (%)	12 (34.3)	6 (25.0)	1.37 (0.60 to 3.15)	0.568
Clinically meaningful improvement, n (%)	31 (88.6)	19 (79.2)	1.12 (0.88 to 1.42)	0.464

Per-protocol population defined as patients who completed all planned induction sessions with a valid end-of-induction PHQ-9 score. As an exploratory analysis, these results are subject to informative dropout bias and are not interpreted as the primary estimate of treatment efficacy.

## 4 Discussion

In this outpatient retrospective observational cohort analysis, both intranasal esketamine and intravenous ketamine were associated with robust antidepressant effects during induction therapy, with no statistically significant difference between groups on any primary or secondary outcome. The magnitude of within-group improvement, with mean PHQ-9 reductions of approximately 9 to 10 points and response rates of 65% to 69%, closely aligns with effect sizes reported in randomized controlled trials and meta-analyses of both agents, supporting external validity of these findings in community psychiatric practice (14–22). However, the modest sample size substantially limits statistical power to detect clinically meaningful between-group differences, and the wide confidence intervals around the between-group comparisons must be interpreted with caution. The absence of a statistically significant between-group difference does not constitute evidence of equivalence, and the present study should not be interpreted as supporting therapeutic interchangeability of these two agents: these findings are best interpreted as descriptive and hypothesis-generating, consistent rather than confirmatory of comparable effectiveness during induction phase treatment.

Our findings should be interpreted in the context of the existing comparative literature, in particular the meta-analysis of Bahji and colleagues (21), which reported greater pooled response and remission rates for intravenous ketamine compared with intranasal esketamine in indirect comparisons of randomized controlled trial data. That analysis was based on between-trial rather than within-study comparisons and has been the subject of methodological discussion in the published literature regarding whether the observed differences reflect drug-specific effects or differences in trial design, patient selection, dosing schedule, and comparator arm. The present study, although underpowered for direct comparison, provides head-to-head observational data from a single community setting using protocols representative of routine clinical practice. Numerical response and remission rates were similar between groups (response 64.9% vs. 69.2%; remission 32.4% vs. 23.1%), and we did not observe the magnitude of separation favoring one therapy over the other in the meta-analytic synthesis. We emphasize that the present study cannot resolve this question given its power limitations; rather, it adds a real-world data point that should be considered alongside the existing-trial based meta-analytic evidence.

Interpretation of treatment effect is strengthened by examining categorical outcomes alongside mean symptom reduction. Response rates were similar between cohorts, indicating that both modalities achieved robust antidepressant effects in the majority of patients. In TRD, where response and remission rates decline markedly with successive traditional treatment failures, this degree of response is clinically substantial and potentially transformative for patients previously without effective options (1). The rate of clinically meaningful improvement was particularly notable: more than 80% of patients treated with intranasal esketamine and more than 70% of those treated with intravenous ketamine achieved at least a 5-point reduction in PHQ-9. Although the difference in clinically meaningful improvement (83.8% vs. 73.1%) did not reach statistical significance, the 10.7 percentage-point separation is the largest numerical difference observed on any binary outcome and may reflect the substantially longer total treatment duration in the esketamine arm (12 sessions over 8 weeks vs. 6 sessions over 3 weeks). The study was underpowered to detect a true difference between modalities at this magnitude, and the finding should be interpreted as hypothesis-generating rather than confirmatory of a between-treatment effect. Our data indicate that the large majority of patients with TRD experience tangible symptomatic benefit during induction therapy even if they did not meet the more stringent thresholds for formal response or remission, an observation of clinical importance given that symptom-burden improvement may translate to better functional outcomes and reduced deterioration requiring hospitalization (1, 23). This is further supported by the within-group BOCF sensitivity analysis showing large treatment effects (Cohen’s d = 1.65 and 1.45 for esketamine and ketamine respectively) even under more conservative assumption regarding those who did not complete therapy.

Remission rates were likewise clinically impressive for a treatment-resistant cohort. Over 32% of intranasal esketamine-treated and 23% of intravenous ketamine-treated patients achieved remission during induction. Whether ongoing maintenance therapy for initial responders would enhance remission rates remains an open question, and a future analysis is planned for this cohort moving forward. The remission rates observed here significantly exceed those historically reported in patients failing multiple standard antidepressant trials, in which remission rates typically fall to low double digits or lower (1). In that context, these observations support the clinical relevance of ketamine-based therapies not only as rapid symptom reducers but as interventions capable of delivering syndromic recovery in a meaningful subset of patients with TRD.

The per-protocol signal reinforces the primary conclusion. Completion rates exceeded 90% in both groups, and outcomes remained similar in completer-only analysis. High completion suggests that these protocols are feasible, generally tolerable, and safe in a structured outpatient practice. Inclusion of clinically relevant non-completers in the primary analysis yields a more conservative and realistic estimate of treatment effect than a purely per-protocol approach, and the BOCF sensitivity analysis demonstrates robustness to the missing-data assumption.

The mechanistic similarity between intravenous ketamine and intranasal esketamine makes the clinical convergence observed here biologically plausible. Both agents exert antidepressant effects through NMDA receptor antagonism and downstream enhancement of synaptic plasticity (24). Differences in route of administration, per-protocol dose, stereochemistry, and pharmacokinetics did not translate into a meaningful difference in short-term antidepressant efficacy within the induction phase within the limits of statistical power afforded by the present sample.

While the study is underpowered for direct comparison, the absence of a clinically meaningful between-group difference shifts the practical focus of treatment selection towards access and implementation. Intranasal esketamine is FDA-approved and commonly covered through commercial insurance or Medicare pathways, albeit with prior authorization requirements and REMS-based administration. Intravenous ketamine for depression remains off-label and is commonly paid for out of pocket. This distinction is highly relevant in community practice: even if two modalities offer similar antidepressant benefit, a therapy that is financially inaccessible to many patients cannot exert the same population-level effect as one with established coverage pathways. The favorable tolerability signal in this cohort also deserves emphasis regarding implications for clinical practice. Discontinuation because of side effects was extremely rare, and no serious adverse events were documented. Although the sample size was not large enough to assess uncommon complications with precision, these findings are directionally consistent with prior reports supporting outpatient feasibility for both modalities when delivered in a structured clinical environment with close monitoring (16, 17, 20).

Several limitations are recognized both in this study’s design and interpretation. The modest sample size (n=37 vs. n=26) substantially limits statistical power and increases the risk of type II error. Wide confidence intervals around between-group estimates preclude conclusions of equivalence, and any apparent between-group similarity should be interpreted with appropriate caution. The retrospective nature of the study introduces the possibility of residual confounding and treatment schedules were not experimentally harmonized, reflecting the intent to study routine practice rather than a controlled trial protocol.

Second, several baseline clinical variables that would be desirable for fuller cohort characterization were not consistently captured in the source EMR in a structured, abstractable format. Specifically, granular counts of prior antidepressant trials beyond the categorical ≥2-failure TRD-eligibility threshold, duration of current depressive episode, exhaustive concomitant medication lists, detailed prior treatment-failure history, and structured psychiatric co-morbidity data were not abstractable for all patients with the ascertainment consistency required to support inferential analysis. We acknowledge that these variables could meaningfully refine cohort characterization in future prospective work. The present analysis is bounded by what the routine clinical record reliably captured, and we chose to report only those baseline variables that were systematically documented across the cohort rather than report incompletely ascertained data that could mislead the reader. Among unmeasured comorbidities, psychiatric conditions such as borderline personality disorder warrant specific mention. Emerging evidence suggests that comorbid personality pathology may differentially modulate response to and tolerability of esketamine relative to racemic ketamine, and differential distribution of such conditions across treatment arms cannot be excluded in this non-randomized cohort (26).

Additionally, the analysis was restricted to induction therapy as well and does not yet address durability of response, maintenance treatment, or relapse prevention, which will be addressed in a planned future analysis. Race and ethnicity were summarized descriptively because sparse subgroup representation precluded meaningful inferential testing. The primary outcome relied on the patient-reported PHQ-9, whereas the comparator randomized trials and the Bahji meta-analysis predominantly employed the clinician-administered Montgomery-Åsberg Depression Rating Scale (MADRS). Direct cross-study comparisons of effect magnitude should therefore be made with caution given established differences between self-reported and clinician-rated instruments in measuring depression severity. Nonetheless, the study has important strengths, including a shared clinical setting, uniform symptom measurement, assurance of TRD diagnosis prior to treatment, and direct real-world comparison of two widely used ketamine-based treatments increasingly employed in community mental health practices.

A central methodological consideration in interpreting between-group comparisons is the structural asymmetry of the two induction protocols. Patients receiving intranasal esketamine completed 12 sessions over 8 weeks, whereas those receiving intravenous ketamine completed 6 sessions over 3 weeks. While both schedules reflect established clinical practice for their respective agents, this asymmetry means that total pharmacological exposure, cumulative dosing, and elapsed time under clinical care differed between groups during induction. The esketamine arm had more than twice the number of treatment contacts, creating potential for greater opportunity for cumulative antidepressant effect, spontaneous improvement with time, and regression to the mean. Conversely, the IV ketamine arm demonstrated numerically comparable PHQ-9 improvement over a substantially compressed timeline, which may reflect a more rapid pharmacodynamic onset per session. These divergent temporal dynamics limit the interpretability of direct endpoint comparisons, and the within-group analysis provides the more interpretable demonstration of antidepressant effect for each agent. Future studies employing time-matched or session-matched analytical designs would meaningfully strengthen comparative inference.

Because this was a non-randomized observational study, treatment assignment was not experimentally controlled and reflects real-world clinical circumstances, including financial considerations as previously mentioned. Consequently, treatment selection in this cohort was almost certainly influenced in part by insurance status and financial resources - unmeasured variables that may be systematically associated with socioeconomic status, health literacy, treatment engagement, and baseline functional capacity, all of which have plausible associations with antidepressant treatment response. While the two groups were well-matched on available baseline characteristics including age, sex, and PHQ-9 severity, residual confounding from unmeasured socioeconomic variables cannot be excluded, and causal interpretation of the between-group comparison should be made with appropriate caution.

A notable strength of the analysis is the heterogeneity of the patient population, which reflects the complexity of real-world TRD more faithfully than the carefully selected populations enrolled in pivotal randomized trials. The observation that response and remission rates in this community cohort closely approximate those seen in controlled trials provides meaningful reassurance that the antidepressant effects of both intranasal esketamine and intravenous ketamine are robust to the real-world clinical complexity. Future studies should focus particularly on dose, duration, and frequency of maintenance therapy to better inform clinical practice guidelines for community providers. Longitudinal studies evaluating durability, relapse rates, and maintenance strategy are especially important. Cost-effectiveness analyses incorporating payer coverage, patient out-of-pocket burden, and downstream healthcare utilization would clarify the economic implications of scaling these therapies. Finally, studies designed to identify predictors of response and remission may enable more personalized selection of patients most likely to benefit from each approach.

Both intranasal esketamine and intravenous ketamine produced substantial antidepressant effects during induction therapy in this community outpatient setting, with large within-group effect sizes (Cohen's d = 1.65 and 1.45 for esketamine and ketamine, respectively, even under conservative BOCF imputation) and high rates of clinically meaningful improvement and remission in a TRD cohort. No statistically significant difference was observed between modalities on any primary or secondary outcome, although the modest sample size substantially limits statistical power and precludes conclusions of equivalence. These findings support the real-world effectiveness of both agents in a community psychiatric practice and suggest that differences in access, coverage, and cost may be more consequential than differences in short-term efficacy when guiding treatment selection for patients with treatment-resistant depression.

## Data Availability

The raw data supporting the conclusions of this article will be made available by the authors, without undue reservation.
